# Editorial: World AIDS Day 2024: take the rights path

**DOI:** 10.3389/fpubh.2026.1859016

**Published:** 2026-05-12

**Authors:** Hailay Abrha Gesesew, Mbuzeleni Hlongwa, Augustine Talumba Choko

**Affiliations:** 1 Research Centre for Public Health Policy, Torrens University, Adelaide, SA, Australia; 2 Tigray Health Research Institute, Mekelle, Ethiopia; 3 School of Health, Medical and Applied Sciences, Central Queensland University, Rockhampton, QLD, Australia; 4 Public Health, Societies and Belonging, Human Sciences Research Council, Pretorial, South Africa; 5 School of Nursing and Public Health Medicine, University of KwaZulu-Natal, Durban, South Africa; 6 Malawi-Liverpool-Welcome Trust Clinical Research Program, Blantyre, Malawi

**Keywords:** AIDS, community advocacy, discrimination, gender equality, HIV, human rights, legal reforms, policy frameworks

World AIDS Day is a moment to reflect on both the achievements and the ongoing challenges of the global HIV response. HIV has been transformed from a fatal diagnosis into a manageable chronic condition, thanks to tremendous biomedical advances—including universal test-and-treat strategies, pre-exposure prophylaxis, and improved antiretroviral therapy. At the same time, as the theme of this Research Topic, *World AIDS Day 2024: Take the rights path*, reminds us, the persistence of new infections and inequities reveals that science alone is not enough. Structural injustice, stigma, criminalization, and social exclusion continue to shape who becomes infected with HIV, who accesses care, and who achieves viral suppression.

To commemorate United Nations World AIDS Day 2024, the Research Topic in *Frontiers in Public Health* entitled “*World AIDS Day 2024: take the rights path*” was launched in 2024, with a team of guest editors overseeing the editorial process to facilitate the timely peer-review and publication of multidisciplinary research contributions addressing rights-based approaches to HIV prevention and care.

A total of 14 manuscripts were submitted of which seven were accepted, representing contributions from 28 authors across multiple global regions. This Research Topic brings together studies spanning Asia, Africa, and North America, with populations including sexually active college/university students, individuals engaged in commercial sexual activities, international migrants from sub-Saharan Africa living with HIV, and healthcare service providers.

Collectively, these contributions highlight how legal frameworks, social norms, institutional practices, and individual perceptions intersect to shape HIV vulnerability and engagement in HIV prevention and care. Across diverse settings—from Chinese universities to South Asian policy environments, from African migrant communities to adolescents living with perinatal HIV in South Africa, and from Canadian service systems to sexually active college populations—the message is consistent: rights-based approaches are foundational to effective HIV prevention and care. By March 2026, this Research Topic had achieved over 15,000 views and more than 3,000 downloads, demonstrating strong global engagement with rights-focused HIV scholarship.

In this Research Topic, the key thematic areas included, but were not limited to:

- Stigma as a persistent structural determinant

*Stigma*—whether rooted in sexuality, migration status, HIV serostatus, drug use, or gender identity—remains a pervasive determinant of risk and care engagement. Its manifestations are psychological, interpersonal, and structural. Addressing stigma requires legal protection, inclusive education, and transformation of institutional cultures. For example, Nyoni and Auslander examine HIV-related stressors and psychological functioning among adolescents with perinatally acquired HIV in KwaZulu-Natal. They demonstrate that stigma experienced by adolescents is significantly associated with lower self-esteem and higher anxiety and depressive symptoms. These findings reinforce that mental health is inseparable from HIV care and underscore the necessity of integrating psychosocial support within routine HIV services.

- *Criminalization and punitive policies undermine public health goals*. Evidence from South Asia and migrant contexts demonstrates that fear of arrest, deportation, or discrimination deters testing and treatment. Decriminalization and anti-discrimination enforcement are not ancillary reforms; they are central to epidemic control. For example, Hoogar's comparative rights-based policy review of HIV responses in India, Nepal, Pakistan, and Sri Lanka demonstrates how criminalization of same-sex conduct, sex work, and drug use undermines progress toward the UNAIDS 95-95-95 targets. Countries that have advanced decriminalization and anti-discrimination protections show stronger outcomes across the HIV cascade and more equitable access to prevention and treatment services.
- *Knowledge alone does not guarantee safer behavior*. The studies among college students reveal that attitudes, perceived barriers, and social environments mediate the translation of knowledge into practice. Rights-based sexuality education must therefore empower young people, respect their autonomy, and provide accessible services. For example, Qi et al. explore the mediating role of attitudes in condom use among sexually active college students. Despite improved knowledge about HIV prevention, a persistent “knowledge–practice gap” remains. Their structural equation modeling demonstrates that perceived benefits and reduced perceived barriers influence condom use primarily through shaping preventive attitudes. This insight reinforces the need for comprehensive sexuality education that not only transmits information but also transforms norms and empowers agency through youth-centered and culturally responsive interventions.
- *Community participation is indispensable*. Whether through migrant-led advocacy, youth-centered campus interventions, or integrated harm reduction services, meaningful engagement of affected populations strengthens both equity and effectiveness.

## Conclusion

We believe that this Edited Research Topic reaffirms that the future of the HIV response depends on integrating biomedical innovation with legal reform, psychosocial support, and structural justice. Furthermore, while we believe that ending AIDS as a public health threat by 2030 remains achievable, it will not be accomplished through treatment scale-up alone given there is also a current financial crisis comprising HIV funding cuts. Rather, it will be achieved when every person—regardless of age, gender, sexuality, migration status, or socioeconomic position—can exercise their right to health without fear and participate meaningfully in shaping the systems designed to serve them.

